# History of a Case of Tic Douloureux, Successfully Treated

**Published:** 1820-05

**Authors:** Andrew Magri

**Affiliations:** Clinical Professor in the University of Ferrara.


FOR THE LONDON MEDICAL AND PHYSICAL JOURNAL.

### *History of a Case of Tic Douloureux, successfully treated.* By Dr. Andrew Magri, Clinical Professor in the University of Ferrara.

A FINE young woman, of a florid complexion, about twenty-two years of age, had suffered, for several years, intense pain in the upper jaw on one side, for which several of her teeth had been extracted, supposing the pain to be seated in those organs. She was tormented during the whole of last winter with spasmodic pains in the left cheek, without any swelling of the affected part, or with only a slight tumefaction about the gums. The spasms would come suddenly, and were extremely acute. The pain seemed to her feelings to be seated in the bones, and darted along the infraorbitary, superior maxillary, and posterior alveolar, nerves; but it was most severe under the zygomatic arcade and above the jugum. The patient had been *ironing* linen for several days before these spasms first appeared, and to the heat of the iron she attributed their origin. She at first received relief from the use of a gargle, made of decoction of sage, barley, and pomegranate rind; and then, in the spring, from one composed of rose-water and laudanum, acidulated with sulphuric acid: but both these soon ceased to afford relief. The pain then extended to the right cheek, and became almost insupportable. The brain and ganglionic nerves became sympathetically affected: hence arose convulsions, irritation of various organs, difficulty of respiration, vergency to delirium, and an abundant evacuation of limpid urine. Mastication was impossible. No topics, no internal remedies, relieved the irritation. Assafœtida, henbane, &c. were of no utility. Recourse was then had to a strong solution of tartar emetic as an external application, partly because I had observed that cold fomentations had somewhat relieved the violence of the pain. The solution produced an evident and unexpected state of calmness of some duration; but the spasms which resembled tic douloureux assumed an exact periodicity, occurring at noon and in the evening. They at length totally ceased under the use of the lotion above described, and fomentations with cold water.

